# A qualitative study on barriers to evidence-based practice in patient counseling and advocacy in Germany

**DOI:** 10.1186/s12913-015-0979-9

**Published:** 2015-08-11

**Authors:** Sibel Altin, Anna Passon, Sibylle Kautz-Freimuth, Bettina Berger, Stephanie Stock

**Affiliations:** Institute for Health Economics and Clinical Epidemiology, University Hospital of Cologne, Gleueler Str. 176-178, 50935 Köln, Germany; Federal Joint Committee (G-BA), Wegelystr. 8, D-10623 Berlin, Germany; Faculty for Health Sciences, University of Witten Herdecke, Center for Integrative Medicine, Gerhard Kienle Institute for Medical Theory, Integrative and Anthroposophic Medicine, Gerhard Kienle Weg 4, D-58313 Herdecke, Germany

## Abstract

**Background:**

Despite the attempt to integrate evidence-based practice (EBP) in patient counseling and advocacy, there is limited knowledge on the status quo of this process in the German health care system. Our objective was to identify important determinants influencing the application of EBP in the counseling and advocacy setting in Germany.

**Methods:**

We carried out a qualitative study performing semi-structured expert interviews and one group discussion among n = 9 patient counselors (PCs) and patient advocates (PAs) identified via expert recommendations and by contacting relevant institutions. The interview manual was developed on the basis of a literature review on barriers/facilitators of EBP in health care delivery and a preamble oriented pyramid discussion with a multidisciplinary team. Interviews were analyzed using the Grounded Theory method. A paradigm was developed to present the interrelations between hindering and facilitating factors for EBP and the attitude towards the utilization of EBP among PAs and PCs.

**Results:**

Findings from nine face-to-face interviews and one group discussion demonstrate that by now PCs and PAs do not recognize EBP as a tool to facilitate the professionalization of patient counselors and advocates. This result is due to individual and institutional barriers such as cognitive-behavioral, professional, attitude related as well as resource and system barriers. PCs and PAs have predominantly critical attitudes towards EBP caused by a lack of trust in its reliability and by concerns regarding unfavorable effects EBP may have on the relationship with the patient and on the cooperation with physicians. A missing infrastructure of needs-based EBP training programs also discourages PCs and PAs from engaging in EBP. Despite the numerous hindering factors, there is also a growing awareness that EBP could help to improve patient counseling and advocacy. To facilitate EBP in future, needs-based training programs and health policy interventions that support interdisciplinary collaboration are required.

**Conclusion:**

Although EBP among PCs and PAs is gaining importance, it is still less likely to be recognized as helpful and its application faces various barriers. More needs-based EBP training programs and health policy interventions to decrease barriers and foster interdisciplinary collaboration are necessary.

**Electronic supplementary material:**

The online version of this article (doi:10.1186/s12913-015-0979-9) contains supplementary material, which is available to authorized users.

## Background

In the last two decades the concept of evidence based medicine (EBM) developed into a gold standard for health services planning and delivery [1]. EBM aims to facilitate the provision of optimal patient care by offering health care decision-making strategies that combine information on the current best medical evidence, cost effectiveness and patient preferences. The implementation of EBM in health care delivery requires evidence based practice (EBP) among health care professionals denoted as the integration of research evidence, clinical expertise and patient values in the daily work processes [2]. To apply EBP successfully health care professionals have to acquire certain skills in obtaining, critically appraising and rapidly incorporating scientific evidence into clinical practice [3]. Although EBM and its practice were originally developed for clinicians, the concept is more and more applied in adjacent fields such as allied health and public health professions [4]. In the context of the German health care system, attempts have been made to integrate EBP in patient counseling and advocacy by training patient counselors and advocates to integrate EBP in their daily work in patient organizations and counseling agencies [5, 6]. The overarching goal is to improve patients’ access to evidence based information, improve their health literacy, and increase their participation in healthcare on all levels [6, 7]. However, there is still paucity of information concerning the status quo of EBP in patient counseling and advocacy in Germany. We neither know whether patient counselors (PCs) and advocates (PAs) are knowledgeable in the basic skills required for the application of EBP, nor do we have sufficient insights into the various factors influencing a successful uptake of EBP among PCs and PAs in Germany. Scientific work on the mechanisms of knowledge translation and the barriers and facilitators for EBP and behavior change demonstrates that there are various factors, which can hinder or facilitate the application of EBP in health care occurring on the individual as well as institutional level [8]. Attempts to organize these multi-faceted factors yielded several models and frameworks for the application of EBP in health care [9–12]. Individual health care professional related barriers and facilitators identified across these models include demographic characteristics (age, gender, motivation, experience), EBP knowledge (cognition, awareness) and skills as well as attitudes towards EBP (ratio, emotions, self-confidence, authority concerns, rigidity of professional boundaries). Institutional factors encompass environmental barriers/facilitators in health care organizations (e.g. resources, time, peer influence, institutional culture) [8–13]. In this regard, individual EBP barriers of health care professionals include a lack of knowledge in and limited awareness of EBM concepts, a rather negative attitude towards EBM methods and EBP strategies, and a lack of self-confidence to perform EBP due to a self-perceived lack of competency and limited sense of authority or responsibility. Examples of institutional barriers include a lack of organizational structures and a lack of human, material and financial resources [8, 10, 13, 14]. The impact of these factors, need to be examined in regard to the role of EBP in patient counseling and advocacy. By now, insights in the perceived hindering and facilitating factors of EBP in patient counseling and advocacy in the German health care context are scarce [5]. To address this knowledge gap, we performed a qualitative study on the perceived barriers and facilitators of EBP in the daily vocational practice of PCs and PAs working in counseling and advocacy facilities in Germany. Our main objective was to identify important determinants influencing the implementation and application of EBP in the counseling and advocacy setting.

## Methods

We carried out a qualitative study performing semi-structured face-to-face expert interviews and one group discussion with patient counselors and patient advocates. Semi-structured interviews combine the advantages of structured and narrative interviews, allowing a well-ordered data collection without limiting the narrative space [15]. The semi-structured interview guide was developed on the basis of the information gained from a literature review on barriers/facilitators of knowledge translation and EBP among health care professionals, particularly considering frameworks and models viewing factors of EBP from the individual and organizational perspective. Three comprehensive models/frameworks for knowledge translation and EBP barriers were identified [9, 10, 13] and served as a basis to develop a list of barrier/facilitator types reported in the literature. To incorporate the interview guide we determined the interview purpose, created a list of EBP barrier-types identified in the previous review and performed a preamble oriented pyramid discussion with an interdisciplinary team (physician, nurse scientist, sociologist). A pyramid discussion is a method that involves raters making choices from a list of items within a given theme or subject [16, 17]. In our case, the pyramid discussion was applied to determine the barrier/facilitator types of EBP relevant for the interview guide and to decide on the content and format of the questions in the guide. Members of the interdisciplinary team of physicians, nurse scientists, and sociologists rated a list of EBP barrier/facilitator types individually, then in pairs, then fours and finally, the whole team took part in a discussion of the ratings. The final version of the interview guide consisted of 15 items in six domains: (1) perceived needs; (2) perceived skills; (3) perceived knowledge gaps; (4) wishes/expectations with regard to formal training classes; (5) facilitators and barriers to formal training classes; (6) socio-demographic variables. A multi-faceted interview guideline allowed for a comprehensive framing of the research question.

### Sample

The convenience sample was chosen from a key target group of PAs and PCs who were identified via expert recommendations and by contacting relevant institutions. Interview participants were identified from the most relevant institutions for counseling, and advocacy in Germany (see Additional file 1). To identify relevant institutions we contacted four counseling and four advocacy experts from academia and public health organizations asking for the most relevant institutions performing patient counseling and advocacy in Germany. These institutions were then contacted via telephone and email. Respondents were classified as PAs if they were not directly involved in counseling individual patients but were active in committee work on various levels of the healthcare system. Respondents were classified as PCs if they were directly involved in counseling individual patients in counseling institutions, self-help groups or patient associations. Participants could be salaried or unsalaried and could be personally affected by a disease or not. Thirty people were classified as PRs or PCs and invited to participate in the semi-structured interviews. Overall, nine face to face interviews and one group discussion were performed. The latter, facilitated the exchange of perceived EBP barriers/facilitators between all nine participants and was performed after all face-to-face interviews were completed. Interviews were continued until category saturation was complete (nine interviews). The interviewer was a trained expert with experience in medical education, qualitative research and EBM. The interviews had an average length of 55 min and were gathered between August and November 2012.

### Ethical considerations

Our research involved only semi-structured interview-guides and de-identified interviews. The interviews were on a voluntary basis and informed written consent was obtained from each participant before data collection. Therefore, the ethical review board of the University Hospital of Cologne did not request an ethical approval.

### Data analysis

**Fig. 1:**
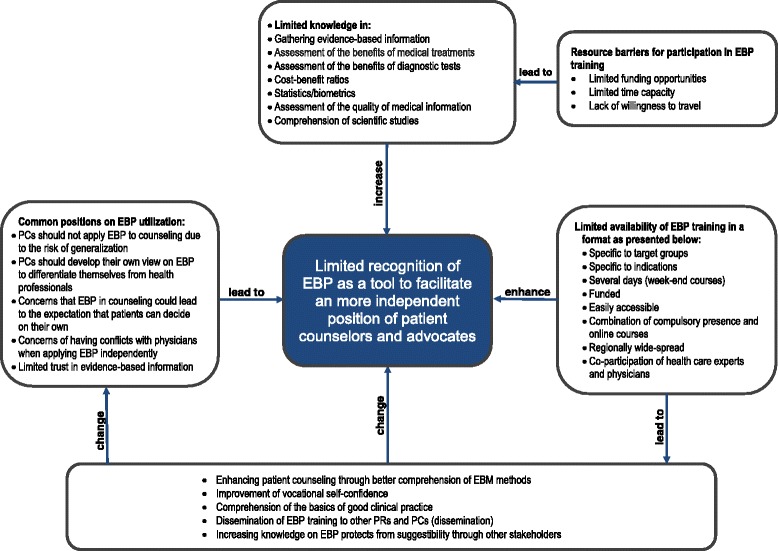
Paradigm of the contextualized determinants affecting PCs and PAs perception of EBP

Interviews were tape-recorded, transcribed verbatim, and analyzed using the Grounded Theory method based on the paradigm of Strauss and Corbin [18]. This method seeks to reduce the material to its essential content in a systematic manner by following a four-step sequence model resulting in a summary of the main statements and a an paradigm. Based on the recommendations of Strauss and Corbin the qualitative data was used to develop a paradigm that presents the interrelations between hindering and facilitating factors for EBP and the attitude towards the utilization of EBP among patient counselors and advocates in the German health care context. To develop the paradigm, the interview statements were divided into several meaning-carrying units and categorized according to different content domains. This was followed by the interpretation of the content domains in regard to their impact on the attitudes of PAs and PCs towards to the utilization of EBP in their daily work, finally yielding the paradigm presented in Fig. 1. This process was performed by two researchers independently to allow for researcher triangulation [19]. Results were included after reaching agreement through discussion between both researchers regarding the interpretation of the data.

## Results

Overall, we performed nine face-to-face interviews and one group interview by using a multi-faceted semi-structured interview guide. The interview sample involved six PAs and three PCs, containing affected patients (four persons) and not affected persons (five persons) as well as voluntary workers (six persons) and salaried staff (three persons) as depicted in Table 1.

**Table 1:** Characteristics of patient counselors and advocates participating in the semi-structured interviews

Inter-viewee	Affected by disease	Patient advocates	Patient counselors
Salaried staff	Not affected	Interview 6: Employee of sickness funds	Interview 3: Counselor of colorectal cancer patients
Salaried staff	Not affected	Interview 2: Medical anthropologist	
Volunteer	Affected	Interview 1: Advocate of prostate cancer patients	Interview 7: Counselor of patients with thyroid diseases
Volunteer	Affected	Interview 5: Advocate of patients with cancer related tumors	Interview 9: Counselor of woman cancer survivors
Volunteer	Not affected	Interview 8: Advocate at the Federal Joint Committee (G-BA)	Interview 4: Employee in a consumer advice center

### Determinants influencing the application of EBP among patient counselors and advocates

The interviews provide insights in the interplay of certain determinants working as barriers and facilitators regarding the implementation of EBP among patient counselors and advocates in the German health care context. Figure 1 displays all identified factors influencing the utilization of EBP in patient advocacy and counseling. A thorough analysis of the qualitative data yields the finding that PCs and PAs do not recognize EBP as a tool to support their vocational professionalization. Even though EBP has the potential to help PCs and PAs to gain independence and improve the way they are recognized or identified as health professionals. According to the data, this negative attitude is due to certain factors working as barriers and facilitators regarding the implementation of EBP in patient counseling and advocacy. In this regard, our analysis demonstrates that individual and institutional barriers affect the attitude of PAs and PCs regarding EBP utilization as presented in Table 2. Individual barriers found are a limited knowledge of EBP (cognitive-behavioral barriers) and a negative attitude towards EBP (professional, attitudinal or rational emotional barriers). Institutional barriers found encompass organizational barriers rooted in the organizational processes (system and process barriers) and the limited availability of resources. These barrier types influence the application of EBP in patient advocacy and counseling through a wide array of mechanisms by interacting with each other or affecting EBP directly. In contrast, we detected certain factors that facilitate the application of EBP among PAs and PCs.

**Table 2:** Detailed contextualization of identified barriers of EBP among patient counselors and advocates

Context factor	Exemplary statement	Definiton	Reference (ID/Page/PC;PA)
Individual barriers to EBP: Professional rational-emotional attitudes as barriers			
Application of EBP	*“Every patient has his individual health problems and psychosocial concerns. In my view, EBP is not specific enough to consider these individual factors.”*	EBP facilitates the risk of generalization when conveyed by medical laypersons	7/9/PC; 9/3/PC
Application of EBP	*“I try to use health related information that are evidence based. Patients often react overwhelmed and feel compelled to decide on their own.”*	EBP places excessive demands on the patient’s competencies and decision making ability	9/1/PC; 8/9/PA
Application of EBP	*“Counseling on evidence based medical informations alone is the task of physicians. We as counselors need to develop our own focus on evidence based counseling.*	PCs should integrate their own view on EBP information	3/1/PC; 9/6/PC
Medical guidelines	*“Guidelines change so quickly and sometimes recommendations are even contradicting. That’s why I do not rely on them.”*	Distrust regarding medical guidelines	7/1/PC; 1/1/PA
Relationship to physicians	*“I think that most counselors are careful when using EBP. The reason is as simple as disappointing! We do not want to have trouble with physicians when applying it.”*	Concerns of having conflicts with physicians when applying EBP	3/3/PC; 7/2/PC; 1/4/PA; 8/2/ PA
	*“In my view most advocates try to deal with EBP and apply it in daily work but at the end most of them follow the opinion of medical specialists. It is so much easier than causing a dispute.”*		
Individual barriers to EBP: Cognitive-behavioral barriers (knowledge and skills)			
Access to EBP information	*“In general, I do support the idea of evidence-based decisions but in my daily practice I cannot even access this information.”*	Gathering evidence-based information on health care issues is difficult.	1/1/PC; 3/1/PC; 7/3/PC; 2/1/PA; 5/2/PA; 6/2/PA
Assessment of benefits in regard to:	*“In my view, the assessment or appraisal of interventions of any kind is the main difficulty I am always faced with and not able to accomplish.”*	The assessment of treatment, diagnostic benefits or cost effectiveness, and information quality of an intervention is very difficult due to complexity of the issue or perceived lack of relevance to daily practice.	Treatment benefit: 1/2/PC; 4/2/PC; 7/4/PC; 5/2/PA
a) diagnostic	*“In EBP one has to consider so many factors and always apply these to the individual case. This sounds easy, but is not.”*		Diagnostic benefit: 4/2/PC; 7/6/PC; 1/2/PA; 6/3/PA; 8/3/PA
b) treatment			
c) cost-effectiveness	*“I never thought about the cost-effectiveness of a treatment. I mean in the end this information does not matter because in most cases we are dealing with interventions that are already paid for by the sickness funds.”*		Cost-effectiveness: 3/2/PC; 4/2/PC; 7/6/PC; 5/2/PA
d) quality of information	*“Off course, I try to critically appraise the quality but in most cases, I just make sure that It is published in a renowned journal because I do not have the skills to access the quality.”*		Information quality: 3/2/PC; 4/3/PC; 7/7/PC; 1/3/PA; 6/4/PA
Individual barriers to EBP: Cognitive-behavioral barriers: (knowledge and skills)			
Statistical skills	*“In most cases, I am not able to comprehend what I am reading. I see odds ratios and p-values without really understanding what it means.”*	Statistical skills are limited	3/2/PC; 4/3/PC; 7/7/PC; 1/3/PA
Methodological skills	*“If you mean the study design I cannot give you an answer because I do not really understand what an evidence grade is and why all studies have to be an RCT. All these aspects are far above what I can apply in my daily work.”*	Limited skills in the concepts of EBM/EBP such as study-design and evidence level	1/2/PA; 6/4/PA
Institutional barriers to EBP: Resource barriers			
Resources	*“To be honest I do not have the time to search for scientific medical papers to use them in my daily work. Unfortunately that’s the truth.”*	Lack of time for EBP	3/3/PC; 4/3/PC; 7/16/PC
Resources	*“As many counselors I’m working on a volunteer basis and cannot afford such a workshop. “*	Limited funding	3/1/PC; 9/6/PC
Resources	*“I cannot travel long distance to participate in such as course.”*	Lack of willingness to travel	9/1/PC; 8/9/PA
Institutional barriers to EBP: System and process barriers			
Participants	*“In my view all professions need a basic understanding of EBP. Programs should be designed for an interprofessional audience.”*	Specific target groups including representatives counselors, physicians and members of self-help groups	3/5/PC; 7/16/PC; 9/9/PC; 4/4/PC; 1/5/PA; 8/3/PA; 6/8/PA; 5/3/PA;
Financial expense	*“In my view, an EBP course should be funded in order to set a strong incentive. Otherwise, only institutions with funding possibilities will be able to send their staff to these courses. In counseling these institutions are scarce.”*	Preference to funding by a third party	3/6/PC; 9/11/PC; 5/3/PA; 8/8/PA
Information dissemination	*„I know that there are EBP programs for laypersons but I never participated because in my area offers are scarce.”*	Programs should be regionally wide-spread and easily assessable.	3/5/PC; 4/5/PC; 7/1/PC; 9/9/PC;
Information dissemination	*“It would be most feasible to have both, presence and online courses to make it more compatible with my job.”*	Programs should combine compulsory presence and online courses	3/5/PC; 4/6/PC; 1/6/PA; 2/5/PA; 8/3/PA

### Individual barriers to EBP in patient counseling and advocacy

#### Professional and attitudinal or rational-emotional barriers

The professional as well as attitudinal or rational-emotional barriers to EBP are similarly recognized by PCs and PAs. It is apparent that there are certain concerns associated with the application of EBP. Respondents emphasize that the use of EBP might overwhelm patients by confronting them with too many complex information and excessively demanding their decision-making abilities. In addition, they fear that a less individualized approach to counseling will develop due to generalizations provided by EBP when applied by laypersons. Respondents also argue that, generally, evidence based counseling is a duty of physicians. They emphasize that, if PCs were to apply evidence based counseling, they would need to integrate their own view on EBP and specifically consider the social background of the patient. In this regard, respondents report that one reason for PCs reluctance to use EBP is the concern to cause discrepancies with physician’s advice given to the patient. This is also reported by PAs affirming that EBP is seen as a specific competence of physicians rather than PAs. As a result PAs indicate to follow the physicians opinion instead of stating the own professional position on EBP. Further, PAs and PCs have a partly critical view on EBM questioning its reliability. This view is based on the opinion that EBP is strongly affected by emerging research findings and new guidelines leading to frequent practice changes, which sometimes contradict prior guidelines and procedures and cause uncertainty. In the majority of cases, PCs and PAs have predominantly critical attitudes towards EBP. This is caused due to a lack of trust in its reliability and concerns regarding unfavorable effects it may have on the relationship with patients and cooperation with physicians. These attitudes contribute to the limited recognition of EBP as a tool to strengthen PCs and PAs unique and independent position.

#### Cognitive-behavioral barriers

Respondents report several cognitive barriers rooted in a limited knowledge of EBM that discourage them from engaging in EBP, ultimately leading to the limited recognition of the benefits of EBP for patient counseling and advocacy as shown in Table 2. They report considerable difficulties when gathering evidence-based information on health care issues or when trying to assess benefits of treatments and diagnostic procedures. Moreover, they emphasize that the assessment of the quality of health related information is difficult to achieve, due to limited understanding of statistics as well as concepts and methods of EBM including effect sizes and level of evidence. A further noteworthy finding is that PCs and PAs do not perceive the cost effectiveness of medical interventions as relevant in their vocational context. In sum, cognitive barriers can have an enormous effect on the acceptance of new concepts. Therefore, novel concepts such as EBM may only be successfully implemented when its basic methodologies are well understood and applied correctly. Otherwise, limited knowledge can cause misjudgments and attitudes of rejection, such as described in the section “attitudinal barriers”.

### Institutional barriers to EBP in patient counseling and advocacy

#### System and process barriers

Participants agree that EBM trainings could help to enhance the role of EBP, but they perceive a lack of infrastructure for adequate EBP training programs that are tailored to the needs of PAs and PCs. Although they report to know of some workshops offered to train PCs and PAs in EBM, they consider these inadequate to meet the vocational needs of the target group. In this regard, PCs and PAs describe their expectations in regard to the design and scope of EBP training programs. For the respondents, a needs-based EBP training should be easily accessible, cover a period of 1 to 2 days, include funding and require both personal class attendance and completion of online courses (Table 2). Furthermore, programs should be tailored to a specific target group, but also include individuals from diverse fields, such as physicians and members of self-help groups. Respondents also recommend a region-wide provision of EBP trainings to increase accessibility. The recommended temporal limitation of the training as well as the preference for funding by a third party and a wide spread provision of trainings may be rooted in a lack of resources in PCs and PAs, who often work on a voluntary basis without financial compensation.

Overall, individual and institutional barriers hamper the application of EBP in patient counseling and advocacy in the German health care context, leading to an underestimation of the benefits of EBP. In contrast, there are some facilitators that are perceived as beneficial for changing the attitudes of PAs and PCs towards the application of EBP.

### Facilitators of EBP application in patient counseling and advocacy

Despite the individual and institutional barriers towards the application of EBP among PCs and PAs, respondents state that there are some factors, which could facilitate the application of EBP in the future. Respondents admit that although they have concerns regarding the effects of EBP on their relationship to patients and other professionals, they concede that knowledge of basic EBM skills could possibly enhance patient counseling by making it more structured and evidence based. Furthermore, they believe that an improvement in methodological competencies could help to strengthen their vocational self-esteem, support professionalization and improve their role functioning, ultimately helping to raise the patient’s voice more effectively. In particular, PAs who are often involved in important reimbursement decisions, report that effective patient advocacy is impossible to achieve without better knowledge of EBM. Indeed, they emphasize that increased professionalization of counselors and advocates, is required to better serve the needs of patients.

## Discussion

This qualitative study aimed to identify and present determinants that influence the application of EBP in patient counseling and advocacy in the German health care context. For this purpose, we carried out semi-structured interviews with nine PCs and PRs, performed a thorough analysis of the qualitative data using the Grounded Theory method and developed a paradigm that describes the effects of barriers and facilitators of EBP on the attitude of PCs and PRs towards the perceived relevance of EBP for patient advocacy and counseling.

Our main finding demonstrates that currently there is a limited recognition of EBP as a tool to establish a unique and independent position for patient counseling and advocacy. This means that PCs and PAs do not perceive EBP as a tool that supports their role as patient advocates and leads to an increased appreciation of their contributions to patient care.

This result may be due to certain determinants that work as barriers regarding the implementation of EBP in patient counseling and advocacy. We identified a comprehensive set of individual and institutional barriers to the application of EBP. Perceived individual barriers are a limited knowledge of EBP (cognitive-behavioral barriers) as well as a negative attitude towards EBP (professional, attitudinal or rational emotional barriers). Institutional barriers identified, encompass organizational barriers rooted in organizational processes (system and process barriers) and the limited availability of resources. These findings are mostly in line with previous research on the barriers of knowledge translation and EBP among health care professionals and allied professions [8, 10–13, 20]. Interestingly, our findings reveal that these barriers also apply to patient counselors and advocates.

The identified barriers significantly influence the limited recognition of the potential benefits of EBP among PCs and PRs. In this regard, prior findings reveal that EBP barriers that interact with each other may have an additive effect [8, 12]. This may also apply to PCs and PAs experiencing several barriers to EBP.

Most important, PCs and PAs have predominantly critical attitudes towards EBP. These are caused by three main reasons: 1. a perceived lack of responsibility for EBP, 2. distrust in the reliability of EBP and 3. concerns regarding unfavorable effects that EBP might have on relationships with patients and cooperation with physicians. These findings are only partly in line with previous research on health care professionals attitudes towards EBP, revealing a rather inconsistent picture of attitudes towards EBP [12, 21, 22]. Especially the concerns regarding unfavorable effects of EBP on the relationship with patients and cooperation with physicians are not reported in the literature, implying that this opinion may be more applicable to the German health care setting.

In this regard, PCs and PAs currently perceive EBP as a skill being more attributable to physicians, thus deciding to not apply EBP in order to avoid confrontation and cooperation problems. This behavior might be due to the deep fragmentation of the German heath care system, which causes a lack of interprofessional collaborations due to strictly differentiated activity profiles and less standardized shared competencies among healthcare professions. Counseling is an essential element of the patient physician relationship and therefore, is traditionally attributed to physicians [2]. Physician representatives such as the National Association of Statutory Health Insurance Physicians (KBV) only reluctantly support a partly delegation of services such as counseling [23]. As a result, the role of patient counseling by PCs is often limited to the provision of health care related information, without recommendations on the eligibility of medical interventions or support in the decision-making process. Thus, the limited sense of responsibility for EBP might be rooted in professional barriers due to concerns of legal issues and the rigidity of professional boundaries often occurring in the German health care context. Changing these factors necessitates health policy interventions that go beyond the delivery of EBM trainings. It is rather required to focus more on the reorganization of service delivery in a way that pool competencies and regulated delegation options may be developed, as recommended by the German Advisory Council of the Ministry of Health [24]. By now, training formats on EBM targeting PAs and PCs are scarce [6, 25] and evidence based patient counseling is limited in practice [16]. This is supported by the findings from our interviews. Among the identified resource, system, and process barriers, it was noted that although PCs and PAs perceive training in EBP as a purposeful way to better understand basic EBM concepts, they judge currently available programs as insufficiently aligned with their professional needs and resources. For future training they recommend a temporal limitation (1 to 2 days), funding by third parties and a wide spread provision of trainings. Following these pieces of advice could help making EBM trainings more acceptable and feasible for PCs and PAs in Germany.

Besides the multiple barriers to EBP among PCs and Pas, we also identified a set of facilitators indicating a possible shift in the overall awareness of EBP and its benefits. For example, respondents recognize that EBP in counseling and advocacy could enhance patient counseling and help strengthening PCs and PAs vocational self-esteem to enable a more professional patient advocacy. Prior studies support this finding, reporting that one major facilitator of EBP is the belief of health care professionals that EBP might have a positive impact on the clinical process and patient outcomes [26]. An improvement in patient counseling and advocacy would be valuable for the German healthcare system, since it proceeds to a more patient-centered system, strengthening patient involvement by increasing the value of patient information, counseling and advocacy [27]. These changes will continuously increase the necessity to equip PAs and PCs with knowledge that makes them less prone to the opinion leadership of other stakeholders [28]. In this regard, elements of EBM, such as critically appraising and communicating scientific evidence, are seen as essential skills [6]. Although there are many hindering factors, there is a growing awareness among PCs and PAs that EBP could serve as a certain component to improve contemporary patient counseling and advocacy when delivered in needs-based formats. This may be seen as the first step in the right direction, but it requires more sophisticated EBP programs and health policy actions to bridge the existing profession barriers.

Our study has some limitations that need to be discussed. First, we used a rather small convenient sample in order to perform more in depth interviews that allow for multi-level analysis. In this regard, a further qualitative study with a larger group of PCs and PRs would be useful to verify our findings. Further, we concentrated on a target group that was familiar with EBM trainings to gauge the importance of educational programs for EBM practice change. Although this approach provided novel insights in the evaluation of currently offered EBP programs a sample without any prior experiences in EBP could have reported greater difficulties in EBP knowledge.

## Conclusion

The German healthcare system is transforming into a more patient-centered system by increasing the value of patient information, counseling and advocacy. This development necessitates sophisticated skills in critically appraising and appropriately communicating medical information among PCs and PAs, thus requiring knowledge in EBP. By now, EBP is not fully recognized as helpful by patient counselors and advocates in the German health care system. This is due to attitudinal, cognitive as well as resource and system barriers that determine a rather negative attitude of PCs and PAs towards EBP. An improvement may be achieved by offering EBM training programs that consider needs as well as resources of PCs and PAs and help promote cognitive skills and reduce attitudinal barriers. Considerable attention must be given to the existing professional barriers that require health policy action such as the implementation of more standardized shared competencies among the healthcare professions.

## Additional file

Additional file 1:
**Key target organizations for the recruitment of patient counselors and representatives.** (DOCX 14 kb)

## Abbreviations

EBM: Evidence based Medicine
EBP: Evidence based Practice
PC: Patient counselor
PA: Patient advocate
KBV: Federal Association for Statutory Health Insurance Physicians

## References

[1] Sackett DL, Rosenberg WM, Gray JA, Haynes RB, Richardson WS (1996). Evidence based medicine: what it is and what it isn’t. BMJ.

[2] Sackett DL, Straus SE, Richardson WS, Rosenberg W, Haynes RB (2000). Evidence-Based Medicine: How to Practice and Teach EBM.

[3] Sackett DL, Rosenberg WM (1995). The Need for Evidence-Based Medicine. J R Soc Med.

[4] Jacobs JA, Jones E, Gabella BA, Spring B, Brownson RC (2012). Tools for Implementing an Evidence-based Approach in Public Health Practice. Prev Chrinic Dis.

[5] Berger B, Gerlach A, Groth S, Sladek U, Ebner K, Mühlhauser I (2013). Competence training in evidence-based medicine for patients, patient counsellors, consumer representatives and health care professionals in Austria: a feasibility study. Z Evid Fortbild Qual Gesundhwes.

[6] Berger B, Steckelberg A, Meyer G, Kasper J, Mühlhauser I (2010). Training of patient and consumer representatives in the basic competencies of evidence-based medicine: a feasibility study. BMC Med Educ.

[7] Dennis S, Williams A, Taggart J, Newall A, Denney-Wilson E, Zwar N (2012). Which providers can bridge the health literacy gap in lifestyle risk factor modification education: a systematic review and narrative synthesis. BMC Fam Pract.

[8] Solomons NM, Spross JA (2011). Evidence-based practice barriers and facilitators from a continous quality improvement perspective: an integrative review. J Nurs Manag.

[9] Grol R, Wensing M (2004). What drives change? Barriers to and incentives for achieving evidence-based practice. Med J Aust.

[10] Cabana MD, Rand CS, Powe NR, Wu AW, Wilson MH, Abboud PA, et al. Why don’t physicians follow clinical pracitce guidelines? A frameworj for improvement. JAMA. 1999;282:1458–65. 10.1001/jama.282.15.1458 10535437

[11] Straus SE, Tetroe JM, Graham ID (2011). Knowledge translation is the use of knowledge in health care decision making. J Clin Epidemiol.

[12] Gray M, Joy E, Plath D, Webb SA (2012). Implementing evidence-based practice: A review of the empirical research literature. Res Soc Work Pract.

[13] Cochrane LJ, Olson CA, Murray S, Dupuis M, Tooman T, Hayes S (2007). Gaps between knowing and doing: Understanding and assessing the barriers to optimal health care. J Contin Educ Health Prof.

[14] Lang ES, Wyer PC, Haynes B (2007). Knowledge translation: Closing the Evidence-to-Practice Gap. Ann Emerg Med.

[15] Stuckey HL (2013). Three types of interviews: Qualitative research methods in social health. J Soc Health Diab.

[16] Blanck B (2005). [Dealing with diversity and alternatives as a challenge for research, teaching and practice]. Umgang mit Vielfalt und Alternativen als Herausforderung für Forschung, Lehre und Praxis. Erwägen-Wissen-Ethik.

[17] Jordan RR (1990). Pyramid discussion. ELT J.

[18] Strauss A, Corbin J (1998). Basics of qualitative research: Techniques and procedures for developing grounded theory.

[19] Mathison S. Why Triangulate? Educ Res. 1988;17:13–7.

[20] Brown CE, Wickline MA, Ecoff L, Glaser D (2009). Nursing practice, knowledge, attitudes and perceived barriers to evidence-based practice at an academic medical center. J Adv Nurs.

[21] Hart P, Eaton L, Buckner M, Morrow BN, Barrett DT, Fraser DD, et al. Effectiveness of a computer-based educational program on nurses’ knowledge, attitude, and skill level related to evidence-based practice. Worldviews Evid Based Nurs. 2008;5:75–84. 10.1111/j.1741-6787.2008.00123.x 18559020

[22] Majid S, Foo S, Luyt B, Zhang X, Theng YL, Chang YK, et al. Adopting evidence-based practice in clinical decision making: nurses’ perceptions, knowledge, and barriers. J Med Libr Assoc. 2011;99:229–36. 10.3163/1536-5050.99.3.010 PMC3133901 21753915

[23] Federal Association for Statutory Health Insurance Physicians (KBV). Resolution on delegation. 2012 http://www.bundesaerztekammer.de/fileadmin/user_upload/downloads/24022012_-_Resolution_Verbaendegespraech.pdf Accessed 12 February 2014.

[24] Sachverständigenrat für die konzentrierte Aktion im Gesundheitswesen (2007). [Cooperation and responsibility - Conditions of a goal-oriented health care]. Kooperation und Verantwortung - Voraussetzungen einen zielorientierten Gesundheitsversorgung.

[25] Weberschock T, Dörr J, Valipour A, Strametz R, Meyer G, Lühmann D, et al. [Evidence-based medicine teaching activities in the German-speaking area: a survey]. Evidenzbasierte Medizin in Aus-, Weiter- und Fortbildung im deutschsprachigen Raum: Ein Survey. Z Evid Fortbild Qual Gesundhwes. 2013;107:5–12. 10.1016/j.zefq.2012.12.005 23415337

[26] Legare F, Ratte S, Gravel K, Graham ID (2008). Barriers and facilitators to implementing shared decision-making in clinical practice: update of a systematic review of health professionals’ perceptions. Patient Educ Couns.

[27] Horch K, Hintzpeter B, Dierks M (2012). [Citizen and patient participation in healthcare. Selected results of the study German GEDA Study.] Bürger- und Patientenorientierung im Gesundheitswesen. Ausgewählte Ergebnisse der GEDA Studie 2009. Bundesgesundheitsblatt Gesundheitsforschung Gesundheitsschutz.

[28] Gigerenzer G, Gaissmaier W, Kurz-Milcke E, Schwartz LM, Woloshin S (2008). Helping doctors and patients make sense of health statistics. Psychol Sci Public Interest.

