# Supplementary material for: A qualitative study on barriers to evidence-based practice in patient counseling and advocacy in Germany
Source: BMC Health Serv Res. 2015 Aug 11;15:317. doi: 10.1186/s12913-015-0979-9 (PMC4542047; doi:10.1186/s12913-015-0979-9)
Supplement: Additional file 1: — Key target organizations for the recruitment of patient counselors and representatives. (DOCX 14 kb) [file 12913_2015_979_MOESM1_ESM.docx]

Appendix

| **Appendix 1:** Key target organizations for the recruitment of patient counselors and representatives | | |
| --- | --- | --- |
| **Target-group** | **Definition** | **Organization** |
| **Patient representative** | Patient advocates generally have no direct contact with patients and represent the patient’s interests in various bodies of the health care system. They can work by main occupation or on a voluntary basis. | - German Disability Council (DBR) - Federal Association of the patients bodies and initiatives (BAGP) - German Association of Self-help groups (DAG SHG) - Federal Association of Self Help (BAG Selbsthilfe) - Federal Joint Committee (G-BA) - Federal office for quality assurance in inpatient care ( BQS) |
| **Patient counselor** | Patient consultants have direct contact with patients and advise patients in counseling facilities, self-help organizations and patient associations either by phone or in a self-help group. Patient **counselors can work on a paid or on a voluntary basis.** | - Independent Patient Counselling Germany (UPD) - Agency for Quality in Medicine (ÄZQ) - Social Association (Sozialverband VdK) - Federation of German Consumer Organizations (vzbv) |
| **Multiplier** | Multipliers disseminate information and opinions in the networks of patient counselors and patient advocates. They can, but do not **have to act as a patient advocate or patient advisor. They are mostly working for sickness** funds or physicians associations. | - Umbrella association of the statutory health insurance (GKV-Spitzenverband) |
